# The genome sequence of the Mauritius kestrel, *Falco punctatus *(Temminck, 1821)

**DOI:** 10.12688/wellcomeopenres.22452.1

**Published:** 2024-06-13

**Authors:** Hernán E. Morales, Ken Norris, Sion Henshaw, Vikash Tatayah, Kevin Ruhomaun, Cock van Oosterhout, Jim J. Groombridge, M. Thomas P. Gilbert, Wellcome Sanger Institute Tree of Life Management, Samples and Laboratory team, Wellcome Sanger Institute Scientific Operations: Sequencing Operations, Wellcome Sanger Institute Tree of Life Core Informatics team, Tree of Life Core Informatics collective

**Affiliations:** 1 Globe Institute, University of Copenhagen, Copenhagen, Denmark; 2 Centre for Evolutionary Hologenomics, University of Copenhagen, Copenhagen, Denmark; 3 Natural History Museum, London, England, UK; 4 Mauritian Wildlife Foundation, Vacoas, Mauritius; 5 National Parks and Conservation Service (Government of Mauritius), Reduit, Mauritius; 6 School of Environmental Sciences, University of East Anglia, Norwich, England, UK; 7 Durrell Institute of Conservation and Ecology, Division of Human and Social Sciences, University of Kent, Canterbury, England, UK

**Keywords:** Falco punctatus, Mauritius kestrel, genome sequence, chromosomal, Falconiformes

## Abstract

We present a genome assembly from an individual male *Falco punctatus* (the Mauritius kestrel; Chordata; Aves; Falconiformes; Falconidae). The genome sequence is 1,279.3 megabases in span. Most of the assembly is scaffolded into 23 chromosomal pseudomolecules, including the Z sex chromosome. The mitochondrial genome has also been assembled and is 17.34 kilobases in length.

## Species taxonomy

Eukaryota; Opisthokonta; Metazoa; Eumetazoa; Bilateria; Deuterostomia; Chordata; Craniata; Vertebrata; Gnathostomata; Teleostomi; Euteleostomi; Sarcopterygii; Dipnotetrapodomorpha; Tetrapoda; Amniota; Sauropsida; Sauria; Archelosauria; Archosauria; Dinosauria; Saurischia; Theropoda; Coelurosauria; Aves; Neognathae; Falconiformes; Falconidae; *Falco*; *Falco punctatus* Temminck, 1821 (NCBI:txid148596).

## Background

**Figure 1.**
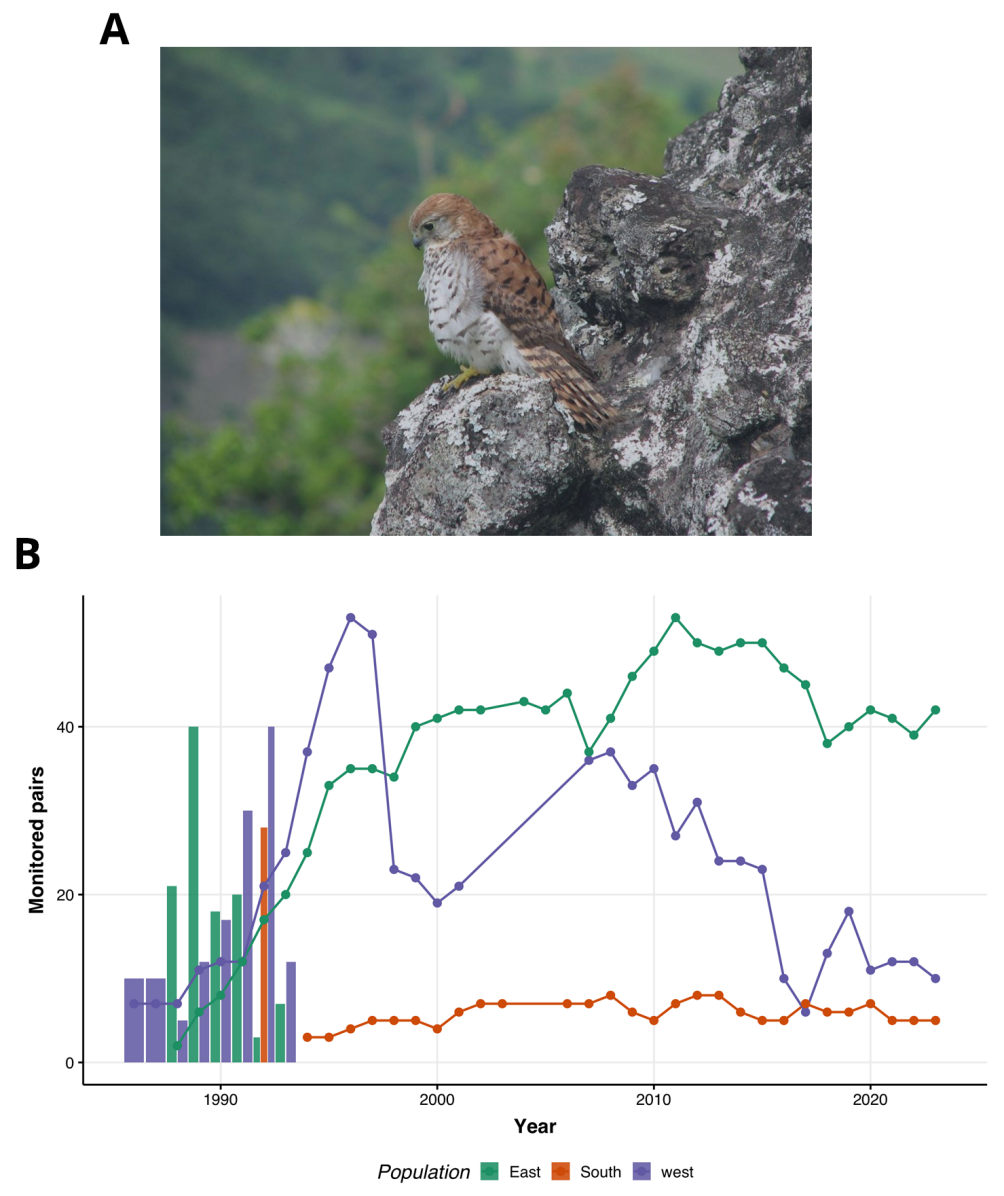
The fall and rise of the Mauritius kestrel. ( **A**) A Mauritius kestrel ( *Falco punctatus;* photo credit Samantha Cartwright) ( **B**) Demographic trajectory over time (bottleneck and recovery), the line represents the number of monitored pairs (territorial pairs) observed each year during the 6-month breeding season *.* The bars represent the number of captive-breed individuals released into the free-living population.

The Mauritius kestrel ( *Falco punctatus*) ( Figure 1A) is a small falcon that is endemic to the island of Mauritius in the Indian Ocean. It is found in forest habitats where it feeds on a range of live caught prey, including endemic geckos, small birds, large insects, and non-native reptiles. The Mauritius kestrel is a territorial species, typically forming monogamous pairs. The birds build their nests in nest boxes or natural cavities in trees and cliffs. Clutches (2 to 5 eggs) are laid from early September onwards, and the last fledglings have usually left the nest by the following January or February. Mauritius kestrels fledge at around 35 days old, achieve independence at around 85 days, and are capable of breeding at 1 year of age. The Mauritius kestrel is usually single brooded but will lay a replacement clutch if its eggs or chicks are lost relatively early in the breeding season.

Prior to the first human settlements in Mauritius in the 1600s, the Mauritius kestrel was widely distributed in forest habitats across the island. By the 1950s, it was restricted to the remote Black River Gorges region due mainly to extensive forest loss and the widespread application of organochlorine and organophosphate pesticides. By 1974, the population had been reduced to only four known wild individuals, making the Mauritius kestrel one of the rarest birds in the World at that time ( Jones *et al.*, 1995).

Despite many conservationists believing the species was doomed to extinction, an intensive conservation programme began in the late 1970s and led to a significant recovery in kestrel numbers and distribution ( Jones *et al.*, 1995; Nicoll *et al.*, 2021) ( Figure 1B). This programme included the management of the remnant wild population through nest box provision, supplementary feeding, and predator control, together with the supplementation of numbers in the wild through captive breeding and release. In response, Mauritius kestrel numbers grew to 350–400 individuals by 2000, and the species was downlisted to Vulnerable on the IUCN Red List. Today, the Mauritius kestrel is found in three main populations, and consists of ~248 individuals. Globally, the recovery of the Mauritius kestrel population is regarded as an iconic conservation success story.

These conservation activities have been accompanied by an ongoing, intensive monitoring programme ( Nicoll *et al.*, 2003). During each breeding season, attempts are made to find as many Mauritius kestrel nests as possible to collect data on the timing of egg laying, the number of eggs laid, and the number of chicks fledged. Prior to fledging, chicks are ringed with a unique combination of coloured leg rings and a numbered metal ring. This has enabled the identification of individual adults returning to breed in subsequent years, and over time has built up an incredibly detailed picture of the lives of individual birds as the populations have recovered. These data have generated a range of unique ecological insights ( Cartwright *et al.*, 2014; Nevoux *et al.*, 2013; Nicoll *et al.*, 2006; Taylor *et al.*, 2021), as well as helping assess and manage ongoing and future risks to the recovering populations ( Nicoll *et al.*, 2021).

The extreme historical population collapse, and the more recent population decline, suggest that the long-term viability of the species might be at risk of a high realised load of harmful deleterious variation ( Dussex *et al.*, 2023). The extensive sample archive and deep ecological knowledge of the species makes it a model study system for conservation genomics. Currently, hundreds of whole genomes are being re-sequenced from historical (pre-1900), recent (1990–2000) and contemporary samples to elucidate the genomic consequence and long-term implications of the historic bottleneck and the more recent population size decline. This research efforts are part of a collaboration between several universities (University of Kent, UK, University of East Anglia, UK, Copenhagen University, Denmark), the Natural History Museum London, the Government of Mauritius’ National Parks and Conservation Service (NPCS) and the Mauritian Wildlife Foundation (MWF - conservation NGO, Mauritius). The conservation monitoring and management of the Mauritius Kestrel is conducted by the MWF in collaboration with the NPCS with guidance from the university partners; recent conservation actions have also been implemented by Ebony Forest Reserve (conservation NGO).

## Genome sequence report

The genome was sequenced from blood sampled from a *Falco punctatus* collected from Bambou Mountains, Mauritius (–20.39, 57.45). A total of 39-fold coverage in Pacific Biosciences single-molecule HiFi long reads was generated. Primary assembly contigs were scaffolded with chromosome conformation Hi-C data. Manual assembly curation corrected 25 missing joins or mis-joins and removed one haplotypic duplication, reducing the scaffold number by 3.36%, and increasing the scaffold N50 by 0.44%.

**Figure 2.**
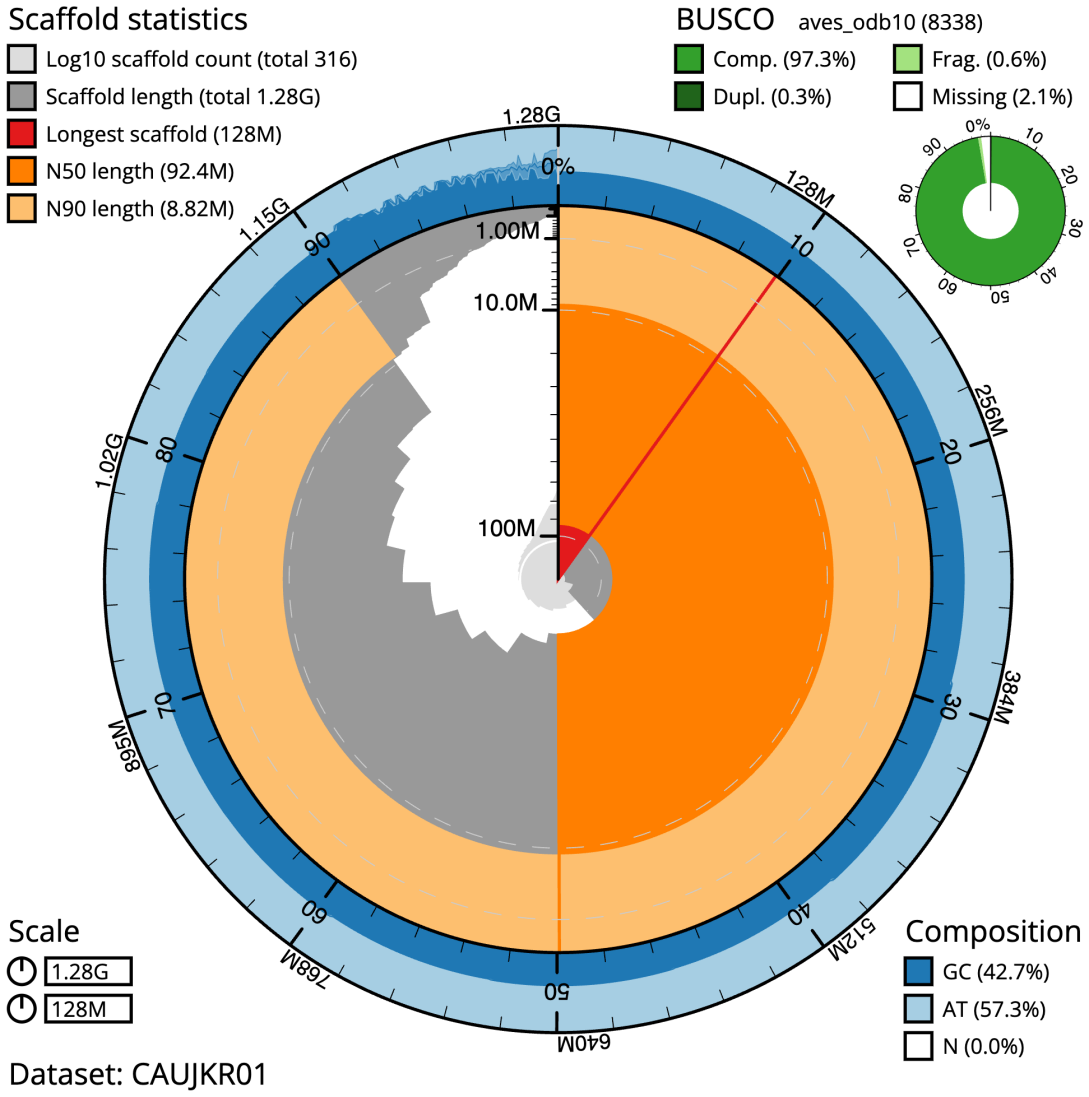
Genome assembly of *Falco punctatus*, bFalPun1.1: metrics. The BlobToolKit snail plot shows N50 metrics and BUSCO gene completeness. The main plot is divided into 1,000 size-ordered bins around the circumference with each bin representing 0.1% of the 1,279,277,844 bp assembly. The distribution of scaffold lengths is shown in dark grey with the plot radius scaled to the longest scaffold present in the assembly (127,704,412 bp, shown in red). Orange and pale-orange arcs show the N50 and N90 scaffold lengths (92,385,878 and 8,824,250 bp), respectively. The pale grey spiral shows the cumulative scaffold count on a log scale with white scale lines showing successive orders of magnitude. The blue and pale-blue area around the outside of the plot shows the distribution of GC, AT and N percentages in the same bins as the inner plot. A summary of complete, fragmented, duplicated and missing BUSCO genes in the aves_odb10 set is shown in the top right. An interactive version of this figure is available at https://blobtoolkit.genomehubs.org/view/Falco%20punctatus/dataset/CAUJKR01/snail.

**Figure 3.**
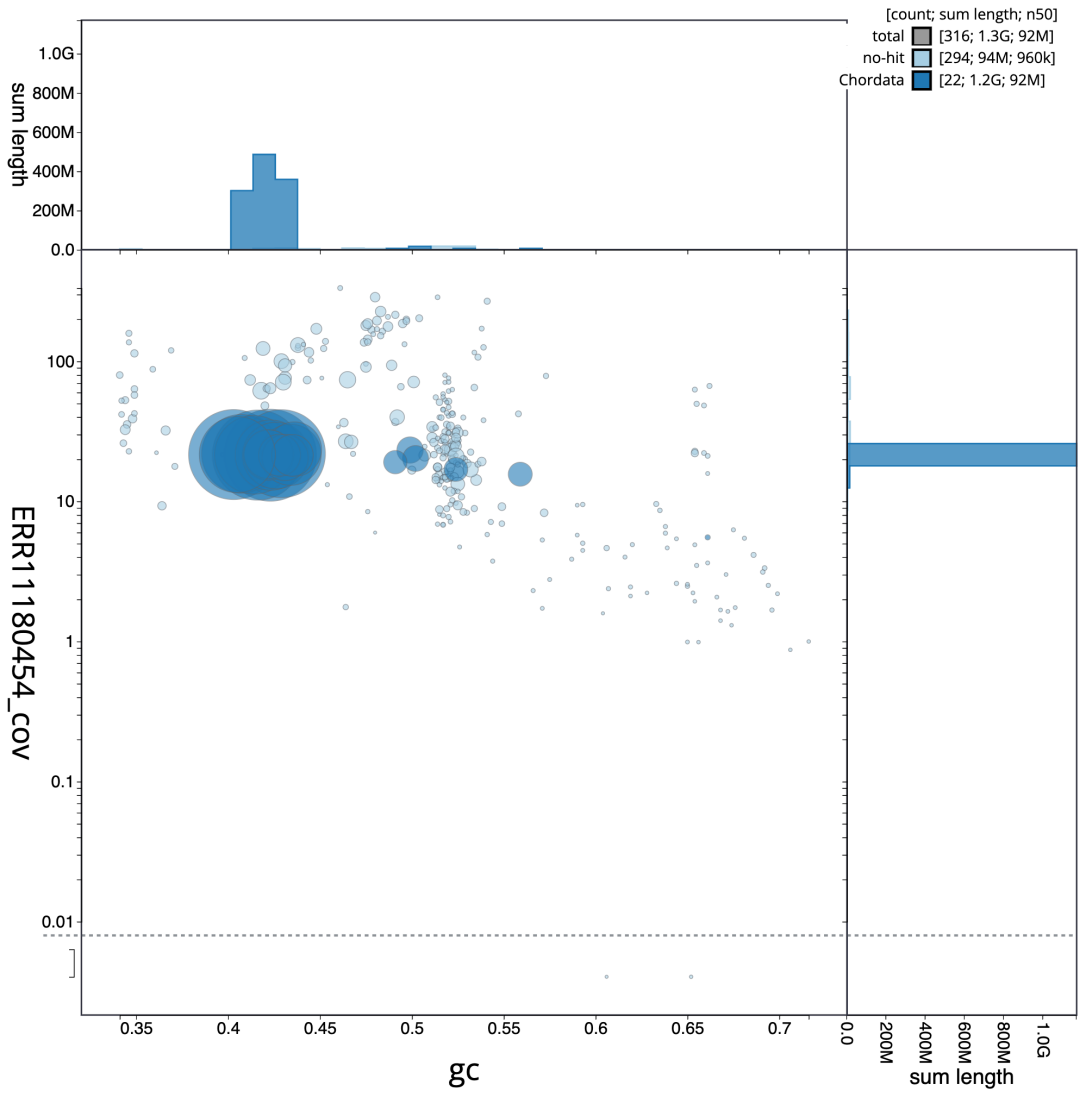
Genome assembly of *Falco punctatus*, bFalPun1.1: BlobToolKit GC-coverage plot. Sequences are coloured by phylum. Circles are sized in proportion to sequence length. Histograms show the distribution of sequence length sum along each axis. An interactive version of this figure is available at https://blobtoolkit.genomehubs.org/view/Falco%20punctatus/dataset/CAUJKR01/blob.

**Figure 4.**
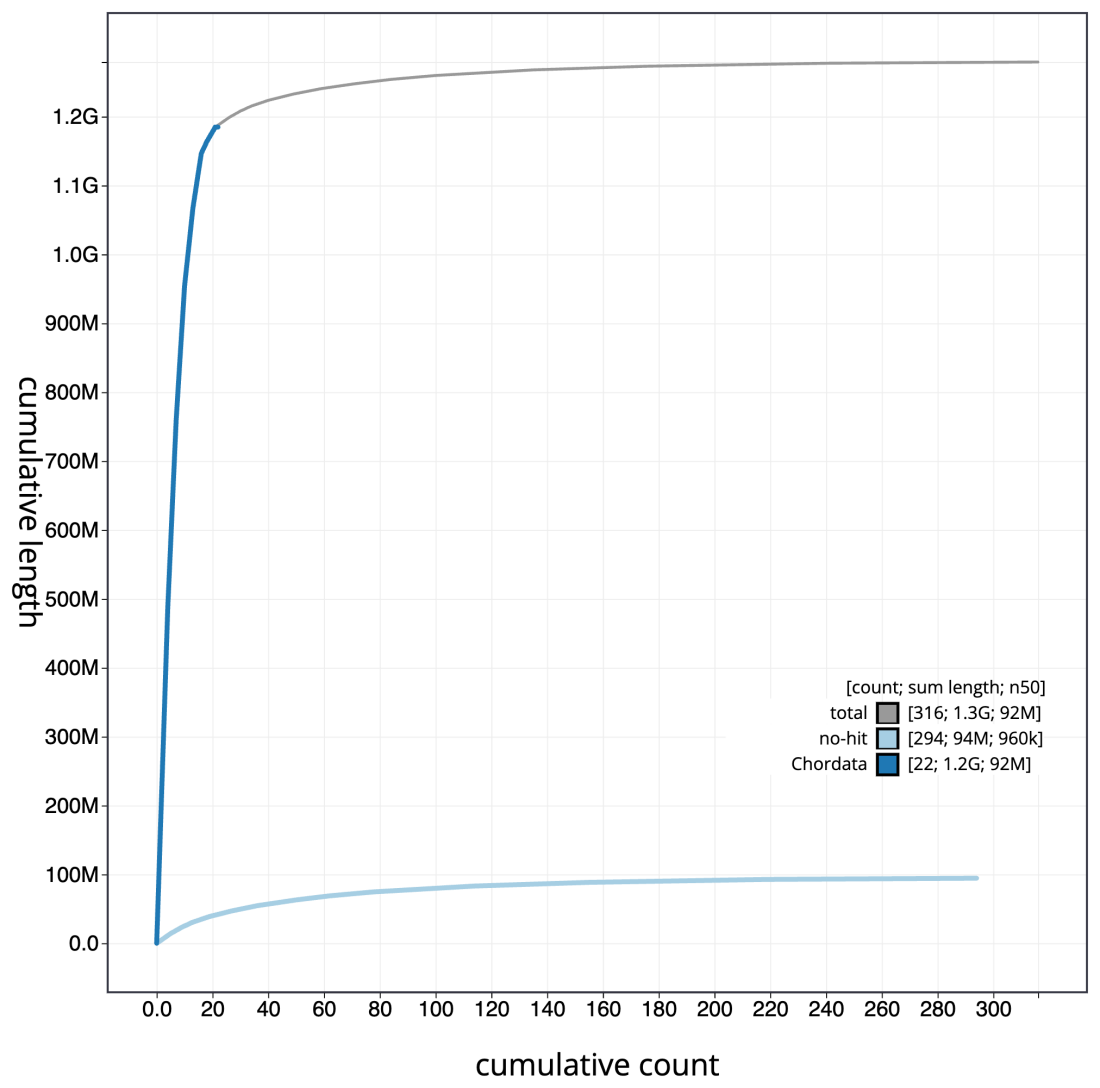
Genome assembly of *Falco punctatus*, bFalPun1.1: BlobToolKit cumulative sequence plot. The grey line shows cumulative length for all sequences. Coloured lines show cumulative lengths of sequences assigned to each phylum using the buscogenes taxrule. An interactive version of this figure is available at https://blobtoolkit.genomehubs.org/view/Falco%20punctatus/dataset/CAUJKR01/cumulative.

**Figure 5.**
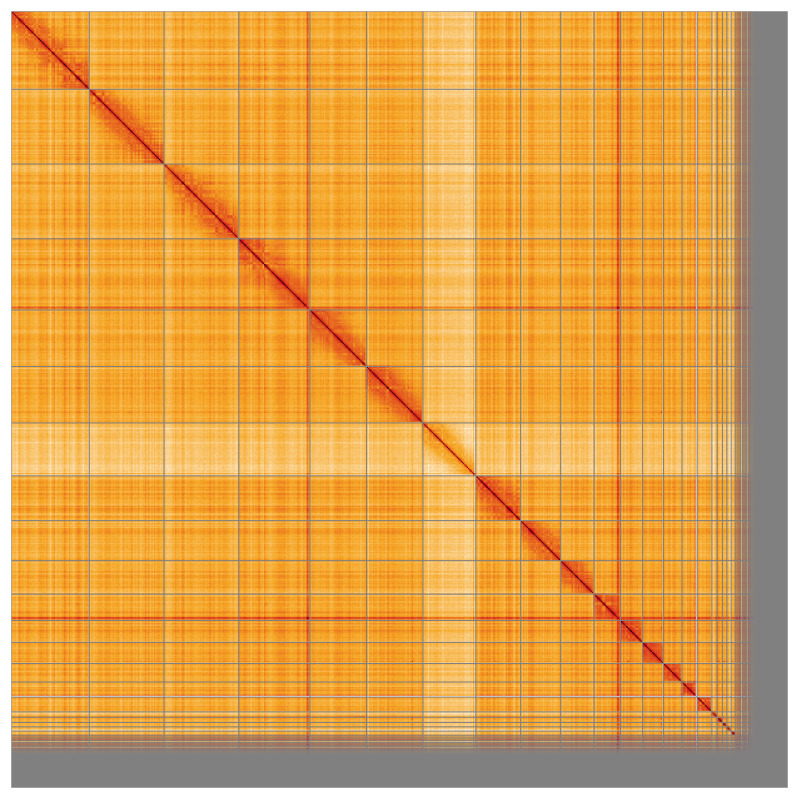
Genome assembly of *Falco punctatus*, bFalPun1.1: Hi-C contact map of the bFalPun1.1 assembly, visualised using HiGlass. Chromosomes are shown in order of size from left to right and top to bottom. An interactive version of this figure may be viewed at https://genome-note-higlass.tol.sanger.ac.uk/l/?d=J1IV79vMQyOL7YmXGxz9LA.

The final assembly has a total length of 1,279.3 Mb in 315 sequence scaffolds with a scaffold N50 of 92.4 Mb ( Table 1). The snail plot in Figure 2 provides a summary of the assembly statistics, while the distribution of assembly scaffolds on GC proportion and coverage is shown in Figure 3. The cumulative assembly plot in Figure 4 shows curves for subsets of scaffolds assigned to different phyla. Most (92.98%) of the assembly sequence was assigned to 23 chromosomal-level scaffolds, representing 22 autosomes and the Z sex chromosome. Chromosome-scale scaffolds confirmed by the Hi-C data are named in order of size ( Figure 5; Table 2). Chromosome Z was assigned by synteny to *Falco biarmicus* (GCF_023638135.1). While not fully phased, the assembly deposited is of one haplotype. Contigs corresponding to the second haplotype have also been deposited. The mitochondrial genome was also assembled and can be found as a contig within the multifasta file of the genome submission.

**Table 1.** Genome data for *Falco punctatus*, bFalPun1.1.

Project accession data		
Assembly identifier	bFalPun1.1	
Species	*Falco punctatus*	
Specimen	bFalPun1	
NCBI taxonomy ID	148596	
BioProject	PRJEB61047	
BioSample ID	SAMEA14356472	
Isolate information	bFalPun1, blood (PacBio DNA and Illumina RNA sequencing) bFalPun2, blood (Illumina Hi-C sequencing)	
Assembly metrics *		*Benchmark*
Consensus quality (QV)	66.8	*≥ 50*
*k*-mer completeness	100.0%	*≥ 95%*
BUSCO **	C:97.3%[S:97.0%,D:0.3%],F:0.6%,M:2.1%,n:8,338	*C ≥ 95%*
Percentage of assembly mapped to chromosomes	92.98%	*≥ 95%*
Sex chromosomes	ZZ	*localised homologous pairs*
Organelles	Mitochondrial genome: 17.34 kb	*complete single alleles*
Raw data accessions		
PacificBiosciences Sequel IIe	ERR11180454, ERR11180453	
Hi-C Illumina	ERR11182529	
PolyA RNA-Seq Illumina	ERR11182528	
Genome assembly		
Assembly accession	GCA_963210335.1	
*Accession of alternate haplotype*	GCA_963210275.1	
Span (Mb)	1,279.3	
Number of contigs	723	
Contig N50 length (Mb)	4.8	
Number of scaffolds	315	
Scaffold N50 length (Mb)	92.4	
Longest scaffold (Mb)	127.7	

* Assembly metric benchmarks are adapted from column VGP-2020 of “Table 1: Proposed standards and metrics for defining genome assembly quality” from Rhie *et al.* (2021). ** BUSCO scores based on the vertebrata_odb10 BUSCO set using version v5.4.3. C = complete [S = single copy, D = duplicated], F = fragmented, M = missing, n = number of orthologues in comparison. A full set of BUSCO scores is available at https://blobtoolkit.genomehubs.org/view/Falco%20punctatus/dataset/CAUJKR01/busco.

**Table 2.** Chromosomal pseudomolecules in the genome assembly of *Falco punctatus*, bFalPun1.

INSDC accession	Chromosome	Length (Mb)	GC%
OY723433.1	1	127.7	42.5
OY723434.1	2	122.35	40.5
OY723435.1	3	122.36	41.5
OY723436.1	4	116.53	43.0
OY723437.1	5	92.39	40.5
OY723438.1	6	92.39	41.5
OY723440.1	7	73.35	42.5
OY723441.1	8	65.51	42.5
OY723442.1	9	54.82	43.5
OY723443.1	10	43.09	43.5
OY723444.1	11	35.83	42.0
OY723445.1	12	34.52	42.0
OY723446.1	13	30.34	43.0
OY723447.1	14	24.6	43.0
OY723448.1	15	24.25	43.5
OY723449.1	16	8.82	50.0
OY723450.1	17	8.47	50.0
OY723451.1	18	7.16	56.0
OY723452.1	19	6.98	52.5
OY723453.1	20	6.74	49.0
OY723454.1	21	1.03	53.5
OY723455.1	22	0.39	55.0
OY723439.1	Z	86.51	40.5
OY723456.1	MT	0.02	46.0

The estimated Quality Value (QV) of the final assembly is 66.8 with *k*-mer completeness of 100.0%, and the assembly has a BUSCO v5.4.3 completeness of 97.3% (single = 97.0%, duplicated = 0.3%), using the vertebrata_odb10 reference set ( *n* = 8,338).

Metadata for specimens, BOLD barcode results, spectra estimates, sequencing runs, contaminants and pre-curation assembly statistics are given at https://links.tol.sanger.ac.uk/species/148596.

## Methods

### Sample acquisition and nucleic acid extraction

Blood sampling of Mauritius kestrels has been conducted by the MWF and university researchers. The Mauritius kestrel population has been blood sampled extensively during restoration; most Mauritius kestrel chicks produced annually were blood sampled, and there has been opportunistic sampling of adults. Chicks are blood sampled in the nest at around 20-days; adults are captured at nest sites for blood sampling. For developing the reference genome, three individuals were captured, and blood samples stored in absolute ethanol at –20°C on 2020-01-29. Blood from specimen with ID SAN11000045 (ToLID bFalPun1) was used for DNA and RNA sequencing, while blood from specimen ID SAN11000046 (ToLID bFalPun2) was used for Hi-C sequencing.

The workflow for high molecular weight (HMW) DNA extraction at the Wellcome Sanger Institute (WSI) Tree of Life Core Laboratory includes a sequence of core procedures: sample preparation; sample homogenisation, DNA extraction, fragmentation, and clean-up. In sample preparation, the bFalPun1 sample was kept on dry ice ( Jay *et al.*, 2023). For sample homogenisation, blood was cryogenically disrupted using the Covaris cryoPREP ^®^ Automated Dry Pulverizer ( Narváez-Gómez *et al.*, 2023). HMW DNA was extracted using the manual Nucleated Blood Nanobind ^®^ protocol ( Denton *et al.*, 2023a). DNA was sheared into an average fragment size of 12–20 kb in a Megaruptor 3 system with speed setting 31 ( Bates *et al.*, 2023). Sheared DNA was purified by solid-phase reversible immobilisation ( Strickland *et al.*, 2023). The concentration of the sheared and purified DNA was assessed using a Nanodrop spectrophotometer and Qubit Fluorometer and Qubit dsDNA High Sensitivity Assay kit. Fragment size distribution was evaluated by running the sample on the FemtoPulse system.

RNA was extracted from a bFalPun1 blood sample in the Tree of Life Laboratory at the WSI using the RNA Extraction: Automated MagMax™ *mir*Vana protocol ( do Amaral *et al.*, 2023). The RNA concentration was assessed using a Nanodrop spectrophotometer and a Qubit Fluorometer using the Qubit RNA Broad-Range Assay kit. Analysis of the integrity of the RNA was done using the Agilent RNA 6000 Pico Kit and Eukaryotic Total RNA assay.

Protocols developed by the WSI Tree of Life laboratory are publicly available on protocols.io ( Denton *et al.*, 2023b).

### Sequencing

Pacific Biosciences HiFi circular consensus DNA sequencing libraries were constructed according to the manufacturers’ instructions. Poly(A) RNA-Seq libraries were constructed using the NEB Ultra II RNA Library Prep kit. DNA and RNA sequencing was performed by the Scientific Operations core at the WSI on Pacific Biosciences Sequel IIe (HiFi) and Illumina NovaSeq 6000 (RNA-Seq) instruments. Hi-C data were also generated from blood sampled from bFalPun2 using the Arima v2 kit. The Hi-C sequencing was performed using paired-end sequencing with a read length of 150 bp on the Illumina NovaSeq 6000 instrument.

### Genome assembly and curation

Assembly was carried out with Hifiasm ( Cheng *et al.*, 2021) and haplotypic duplication was identified and removed with purge_dups ( Guan *et al.*, 2020). The assembly was then scaffolded with Hi-C data ( Rao *et al.*, 2014) using YaHS ( Zhou *et al.*, 2023). The assembly was checked for contamination and corrected as described previously ( Howe *et al.*, 2021). Manual curation was performed using HiGlass ( Kerpedjiev *et al.*, 2018) and PretextView ( Harry, 2022). The mitochondrial genome was assembled using MitoHiFi ( Uliano-Silva *et al.*, 2023), which runs MitoFinder ( Allio *et al.*, 2020) or MITOS ( Bernt *et al.*, 2013) and uses these annotations to select the final mitochondrial contig and to ensure the general quality of the sequence.

### Final assembly evaluation

The final assembly was post-processed and evaluated with the three Nextflow ( Di Tommaso *et al.*, 2017) DSL2 pipelines “sanger-tol/readmapping” ( Surana *et al.*, 2023a), “sanger-tol/genomenote” ( Surana *et al.*, 2023b), and “sanger-tol/blobtoolkit” ( Muffato *et al.*, 2024). The pipeline sanger-tol/readmapping aligns the Hi-C reads with bwa-mem2 ( Vasimuddin *et al.*, 2019) and combines the alignment files with SAMtools ( Danecek *et al.*, 2021). The sanger-tol/genomenote pipeline transforms the Hi-C alignments into a contact map with BEDTools ( Quinlan & Hall, 2010) and the Cooler tool suite ( Abdennur & Mirny, 2020), which is then visualised with HiGlass ( Kerpedjiev *et al.*, 2018). It also provides statistics about the assembly with the NCBI datasets ( Sayers *et al.*, 2024) report, computes *k*-mer completeness and QV consensus quality values with FastK and MerquryFK, and a completeness assessment with BUSCO ( Manni *et al.*, 2021).

The sanger-tol/blobtoolkit pipeline is a Nextflow port of the previous Snakemake Blobtoolkit pipeline ( Challis *et al.*, 2020). It aligns the PacBio reads with SAMtools and minimap2 ( Li, 2018) and generates coverage tracks for regions of fixed size. In parallel, it queries the GoaT database ( Challis *et al.*, 2023) to identify all matching BUSCO lineages to run BUSCO ( Manni *et al.*, 2021). For the three domain-level BUSCO lineage, the pipeline aligns the BUSCO genes to the Uniprot Reference Proteomes database ( Bateman *et al.*, 2023) with DIAMOND ( Buchfink *et al.*, 2021) blastp. The genome is also split into chunks according to the density of the BUSCO genes from the closest taxonomically lineage, and each chunk is aligned to the Uniprot Reference Proteomes database with DIAMOND blastx. Genome sequences that have no hit are then chunked with seqtk and aligned to the NT database with blastn ( Altschul *et al.*, 1990). All those outputs are combined with the blobtools suite into a blobdir for visualisation.

All three pipelines were developed using the nf-core tooling ( Ewels *et al.*, 2020), use MultiQC ( Ewels *et al.*, 2016), and make extensive use of the Conda package manager, the Bioconda initiative ( Grüning *et al.*, 2018), the Biocontainers infrastructure ( da Veiga Leprevost *et al.*, 2017), and the Docker ( Merkel, 2014) and Singularity ( Kurtzer *et al.*, 2017) containerisation solutions.

Table 3 contains a list of relevant software tool versions and sources.

**Table 3.** Software tools: versions and sources.

Software tool	Version	Source
BEDTools	2.30.0	https://github.com/arq5x/bedtools2
Blast	2.14.0	ftp://ftp.ncbi.nlm.nih.gov/blast/executables/blast+/
BlobToolKit	4.3.7	https://github.com/blobtoolkit/blobtoolkit
BUSCO	5.4.3 and 5.5.0	https://gitlab.com/ezlab/busco
bwa-mem2	2.2.1	https://github.com/bwa-mem2/bwa-mem2
Cooler	0.8.11	https://github.com/open2c/cooler
DIAMOND	2.1.8	https://github.com/bbuchfink/diamond
fasta_windows	0.2.4	https://github.com/tolkit/fasta_windows
FastK	427104ea91c78c3b8b8b49f1a7d6bbeaa869ba1c	https://github.com/thegenemyers/FASTK
GoaT CLI	0.2.5	https://github.com/genomehubs/goat-cli
Hifiasm	0.16.1-r375	https://github.com/chhylp123/hifiasm
HiGlass	44086069ee7d4d3f6f3f0012569789ec138f42b84aa44357826c0b6753eb28de	https://github.com/higlass/higlass
MerquryFK	d00d98157618f4e8d1a9190026b19b471055b22e	https://github.com/thegenemyers/MERQURY.FK
MitoHiFi	2	https://github.com/marcelauliano/MitoHiFi
MultiQC	1.14, 1.17, and 1.18	https://github.com/MultiQC/MultiQC
NCBI Datasets	15.12.0	https://github.com/ncbi/datasets
Nextflow	23.04.0-5857	https://github.com/nextflow-io/nextflow
PretextView	0.2	https://github.com/wtsi-hpag/PretextView
purge_dups	1.2.3	https://github.com/dfguan/purge_dups
samtools	1.16.1, 1.17, and 1.18	https://github.com/samtools/samtools
sanger-tol/genomenote	1.1.1	https://github.com/sanger-tol/genomenote
sanger-tol/readmapping	1.2.1	https://github.com/sanger-tol/readmapping
Seqtk	1.3	https://github.com/lh3/seqtk
Singularity	3.9.0	https://github.com/sylabs/singularity
YaHS	yahs-1.1.91eebc2	https://github.com/c-zhou/yahs

### Wellcome Sanger Institute – Legal and Governance

The materials that have contributed to this genome note have been supplied by a Tree of Life collaborator. The Wellcome Sanger Institute employs a process whereby due diligence is carried out proportionate to the nature of the materials themselves, and the circumstances under which they have been/are to be collected and provided for use. The purpose of this is to address and mitigate any potential legal and/or ethical implications of receipt and use of the materials as part of the research project, and to ensure that in doing so we align with best practice wherever possible.

The overarching areas of consideration are:

•      Ethical review of provenance and sourcing of the material

•      Legality of collection, transfer and use (national and international)

Each transfer of samples is undertaken according to a Research Collaboration Agreement or Material Transfer Agreement entered into by the Tree of Life collaborator, Genome Research Limited (operating as the Wellcome Sanger Institute) and in some circumstances other Tree of Life collaborators.

## Data Availability

European Nucleotide Archive: *Falco punctatus* (Mauritius kestrel). Accession number PRJEB61047; https://identifiers.org/ena.embl/PRJEB61047 ( Wellcome Sanger Institute, 2023). The genome sequence is released openly for reuse. The *Falco punctatus* genome sequencing initiative is part of the Vertebrate Genomes Project. All raw sequence data and the assembly have been deposited in INSDC databases. The genome will be annotated using available RNA-Seq data and presented through the Ensembl pipeline at the European Bioinformatics Institute. Raw data and assembly accession identifiers are reported in Table 1.

## Author information

Members of the Wellcome Sanger Institute Tree of Life Management, Samples and Laboratory team are listed here: https://doi.org/10.5281/zenodo.10066175.

Members of Wellcome Sanger Institute Scientific Operations: Sequencing Operations are listed here: https://doi.org/10.5281/zenodo.10043364.

Members of the Wellcome Sanger Institute Tree of Life Core Informatics team are listed here: https://doi.org/10.5281/zenodo.10066637.

Members of the Tree of Life Core Informatics collective are listed here: https://doi.org/10.5281/zenodo.5013541.
