# Supplementary material for: The genome sequence of the Mauritius kestrel, Falco punctatus (Temminck, 1821)
Source: Wellcome Open Res. 2024 Jun 13;9:312. [Version 1] doi: 10.12688/wellcomeopenres.22452.1 (PMC11362723; doi:10.12688/wellcomeopenres.22452.1)
Supplement: Peer review history

# Reviewer report: Reviewer response for version 1

**DOI:** 10.21956/wellcomeopenres.24739.r92412

**Published:** 2024-08-29

**Authors:** Daniel E Chavez

**Affiliations:** 1 Pontifcia Universidad Católica del Ecuador, Quito, Ecuador; 2 Arizona State University, Tempe, AZ, USA; 3 University of California Los Angeles, Los Angeles, California, USA

Clearly written manuscript which is very important for investigating the historical population collapse of the Mauritius kestrel. This study contains sufficient data analysis (e. sanger-tol and bwa) for a high-quality reference genome. Given this manuscript covers several different methods to evaluate the genome (e.g Hi-C contact map), the assumed knowledge is quite high.

 Your manuscript could benefit from a brief clarification about the effect of using two different individuals one for the genome and another for the Hi-C. Also, it is not clear from the manuscript if the aforementioned samples were males. Finally, the evaluation of the annotation could benefit from gene prediction models see for instance Salojärvi J et al.(2024 ^1^ )

Are sufficient details of methods and materials provided to allow replication by others?

Yes

Is the rationale for creating the dataset(s) clearly described?

Yes

Are the datasets clearly presented in a useable and accessible format?

Yes

Are the protocols appropriate and is the work technically sound?

Yes

Reviewer Expertise:

Molecular Biology, Genomics and Bioinformatics.

I confirm that I have read this submission and believe that I have an appropriate level of expertise to confirm that it is of an acceptable scientific standard.

## References

[1] : The genome and population genomics of allopolyploid Coffea arabica reveal the diversification history of modern coffee cultivars. *Nat Genet* .2024;56(4) : 10.1038/s41588-024-01695-w 721-731 10.1038/s41588-024-01695-w 38622339 PMC11018527

# Reviewer report: Reviewer response for version 1

**DOI:** 10.21956/wellcomeopenres.24739.r92413

**Published:** 2024-08-22

**Authors:** Katarina C Stuart

**Affiliations:** 1 The University of Auckland, Auckland, Auckland, New Zealand

This data note by Morales et al., presents the genome sequence of the Mauritius kestrel, *Falco punctatus*. This species is of high conservation concern and has significant inbreeding due to population declines in recent history. Thus, I believe this genome will be an extremely valuable resource for future conservation genomics studies, including the ongoing genome re-sequencing efforts mentioned in the note. The methods of genome assembly and assessment are sound and reflect the methods used in many Tree of Life genome notes. I noticed that this report is missing methods and result for the genome annotation, which I think should be included and released with the main genome assembly. Other than that, I have no further comments and am looking forward to the forthcoming genomics research on this species.

Are sufficient details of methods and materials provided to allow replication by others?

Yes

Is the rationale for creating the dataset(s) clearly described?

Yes

Are the datasets clearly presented in a useable and accessible format?

Yes

Are the protocols appropriate and is the work technically sound?

Yes

Reviewer Expertise:

genome assembly, conservation and evolutionary genomics, invasive species

I confirm that I have read this submission and believe that I have an appropriate level of expertise to confirm that it is of an acceptable scientific standard.
